# Selenium Biotransformation and Fractionation of Selenopeptide from Germinated Perilla (*Perilla frutescens*) Seeds

**DOI:** 10.3390/foods14172988

**Published:** 2025-08-27

**Authors:** Tanaporn Monkhai, Saroat Rawdkuen, Suphat Phongthai, Pornrawin Pakdeebamrung, Naphatsawan Singhadechachai, Apinya Chaikaew, Pornchai Rachtanapun, Pipat Tangjaidee

**Affiliations:** 1 Division of Food Science and Technology, Faculty of Agro-Industry, Chiang Mai University, Chiang Mai 50100, Thailand; tanaporn_monkhai@cmu.ac.th (T.M.); apinya_chaik@cmu.ac.th (A.C.); 2 Innovative Food Science and Technology Program, School of Agro-Industry, Mae Fah Luang University, Chiang Rai 57100, Thailand; 3 Center of Excellence in Agro Bio-Circular-Green Industry (Agro BCG), Chiang Mai University, Chiang Mai 50100, Thailand; 4 Division of Packaging Technology, Faculty of Agro-Industry, Chiang Mai University, Chiang Mai 50100, Thailand

**Keywords:** *Perilla frutescens*, hydrolysis, antioxidant activity, selenopeptides (Se-peptides), selenium, selenite, ACE inhibitory activity

## Abstract

Plant-based bioactive compounds have been recognized as promising alternatives to conventional chemical treatments. Selenium (Se), a trace element, can be incorporated into proteins to enhance the bioactivity of plant-derived peptides. *Perilla frutescens* seeds are high-protein plants that have shown the ability to absorb Se and biosynthesize selenopeptides. This study examined Se biotransformation during the germination of perilla seeds to synthesize selenoprotein, investigating enzymatic hydrolysis using Alcalase and Flavourzyme as single enzymes, as well as their combinations. The results showed that Alcalase hydrolysates produced Se-peptides with the highest degree of hydrolysis and antioxidant activity. Hydrolysates were purified via ultrafiltration and size-exclusion chromatography, and Se-peptides were characterized by LC-MS/MS. Nine peptides containing Se-binding residues such as cysteine, methionine, and glutamic acid confirmed successful Se incorporation. The Se-peptides demonstrated strong antioxidant activity (ABTS: 66.30%, FRAP: 54.93%), ACE inhibition (83.87%), and cytotoxicity against A549 lung cancer cells (85.88% viability). Compared to non-Se-peptides, Se-enriched peptides showed superior bioactivity, highlighting their potential as functional ingredients in food and pharmaceutical applications.

## 1. Introduction

Perilla (*Perilla frutescens* L. Britt.) is an annual herb belonging to the Lamiaceae family. The plant has been traditionally cultivated and used in East and Southeast Asian countries such as China, Korea, Japan, Laos, and Thailand. In northern Thailand (e.g., Chiang Mai, Chiang Rai, and Maehongsorn), it is locally known as nga-kee-mon [1,2]. Historically, perilla has been valued for its diverse uses, including culinary, medicinal, and industrial purposes [3]. Perilla seeds are not only good sources of oil but are also good sources of protein, with content ranging from 18 to 23% (dry weight), depending on cultivar and growing conditions. These proteins exhibit a balanced amino acid profile, providing all essential amino acids except tryptophan (Trp) for adults. The perilla seed protein is rich in acidic amino acids (aspartic acid; Asp and glutamic acid; Glu), contributing to its functional potential, despite a relatively low lysine (Lys) content [4,5].

Plant-derived peptides have recently attracted considerable attention due to their diverse bioactivities, particularly as natural antihypertensive and anticancer agents. Several peptides from plant proteins have demonstrated potent angiotensin I-converting enzyme (ACE) inhibitory activity, helping to regulate blood pressure by preventing the conversion of angiotensin I to the vasoconstrictor angiotensin II and inhibiting bradykinin degradation. In addition, certain bioactive peptides exhibit antiproliferative effects on cancer cells through mechanisms such as the induction of apoptosis, the inhibition of angiogenesis, and the disruption of cellular redox balance [6,7]. These bioactivities make plant peptides promising candidates for the development of functional foods and nutraceuticals.

Selenium (Se) is a vital micronutrient that supports the activity of antioxidant enzymes such as thioredoxin reductase (TrxR) and glutathione peroxidase (GPx), which help neutralize harmful free radicals [8]. Since the body cannot synthesize Se, dietary intake is essential. One effective strategy to increase Se content in food crops is biofortification using inorganic forms such as sodium selenate (SeO_4_^2−^) and sodium selenite (SeO_3_^2−^). During germination, inorganic Se (selenite and selenate) can be converted into organic Se-peptides (selenocysteine (SeCys) and selenomethionine (SeMet)), which are less toxic and more bioavailable. Studies on Se biotransformation in crops like mushrooms, soybean sprouts, chickpeas, and broccoli have shown that Se-peptides can enhance antioxidant capacity [8,9,10,11]. Perilla seeds are suitable for Se enrichment due to their high content of Se-binding amino acids such as cysteine (Cys), methionine (Met), histidine (His), arginine (Arg), Asp, Glu, and Lys [5,12].

The way to produce bioactive peptides is enzymatic hydrolysis. Enzymatic hydrolysis is a widely used method for producing peptides. This approach is popular due to the high substrate specificity of enzymes and their ability to break down proteins under mild conditions [13]. Peptides or protein hydrolysates obtained through this process have the potential to act as natural antioxidants. These hydrolysates can neutralize free radicals by donating hydrogen or electrons, as well as reducing hydroperoxides and reactive oxygen species. Alcalase and Flavourzyme are potential candidates for industrial hydrolysate production. Alcalase, an endopeptidase, cleaves peptide bonds at the C-terminal of Glu, phenylalanine (Phe), leucine (Leu), Lys, tyrosine (Tyr), Trp, and Met amino acids and is known for its high efficiency, resulting in strong antioxidant and ACE inhibitory activity. In contrast, Flavourzyme functions as both an endopeptidase and an exopeptidase, breaking peptide chains at the N-terminal by cleaving the peptide bond between Leu and proline (Pro) or Pro and Pro [14,15]. The biological activity of the resulting peptides is influenced by several factors, including enzyme specificity, the degree of hydrolysis, and the nature of the peptides released, including molecular weight, amino acid composition, and hydrophobicity [16]. Recent evidence also suggests that Se-peptides produced via enzymatic hydrolysis can exert multifunctional effects, including the production of antioxidants, ACE inhibition, and cytotoxic activity against cancer cells [6,7].

Despite growing interest, studies on Se biotransformation, enzymatic hydrolysis, and the biological activities of Se-peptides derived from perilla seeds remain limited. Therefore, the objectives of this study were to investigate Se biotransformation during perilla seed germination, examine the enzymatic hydrolysis of Se-peptide production, and evaluate the antioxidants, ACE inhibition, and anticancer activity of Se-peptide fractions. The results of this work aim to support the application of Se-peptides from perilla seeds in the development of functional foods and therapeutic agents.

## 2. Material and Methods

### 2.1. Materials

Perilla seeds (*Perilla frutescens*) were harvested from the fields in Saraphi district, Chiang Mai province, Thailand, and stored at ambient temperature in a dark room until used. All reagents were of analytical grade and used without further purification. Sodium selenite (Na_2_SeO_3_, 99%), 2,2′-azino-bis (3-ethylbenzothiazoline-6-sulphonic acid) diammonium salt (ABTS, ≥98%), 2,4,6-Tris(2-pyridyl)-s-triazine (TPTZ, ≥99%), and Flavourzyme from *Aspergillus oryzae* (≥500 U/g) were purchased from Sigma-Aldrich (St. Louis, MO, USA). Selenium ICP standard, Alcalase enzyme from *Bacillus Licheniformis* (2.960 U/mL), hydrogen peroxide (H_2_O_2_, 30%), were purchased from Merck (Billerica, MA, Germany). Ferric chloride hexahydrate (FeCl_3_.6H_2_O, 97%) was purchased from LOBA Chemie (Mumbai, India). Boric acid (H_3_BO_3_, 99.5%), hydrochloric acid (HCL, 37%), sulfuric acid (H_2_SO_4_, 98%), and sodium hydroxide (NaOH) from RCI Labscan (Bangkok, Thailand). Other chemicals and solvents of analytical grade were obtained from different commercial sources.

### 2.2. Selenium Enrichment in Perilla frutescens Seeds

The enrichment of Se in perilla seeds was performed by the following method, described by Lintschinger et al. [17], with slight modification. The perilla seeds were first cleaned and soaked in sodium selenite solutions of varying concentrations (20, 40, 60, 80, and 100 ppm) in plastic trays (12 × 20 cm). For the control sample (without Se), distilled water was used in place of the solutions. After soaking overnight, the seeds were rinsed; then, they were washed by adding fresh solutions of the same concentration and left to soak for 10 min before rinsing again. We then spread the seeds evenly in the tray. The seeds were incubated at room temperature (25–27 °C) for one week, with daily washing, until 80% of the roots reached a length of 2–3 cm, to ensure Se absorption. Before sample collection, the seeds were washed three times with distilled water to remove any remaining solutions on the surface. Samples were dried using a hot-air oven at 50 °C for 6 h, ground, defatted two times using petroleum ether (10% *w*/*v*) [18], and stored at −20 °C for further analysis. The fat content in the seeds before they were defatted was analyzed by the Soxtec method [19]. The optimal Se-enrichment conditions were determined based on total protein and Se content. The condition with the highest Se and protein content was selected for future experiments including control samples.

### 2.3. Selenoprotein Extraction and Enzymatic Hydrolyzation

Protein extraction of Se-enriched *Perilla frutescens* seeds was carried out using an alkalization/precipitation procedure following a modified procedure by Kim et al. [20]. The defatted Se-enriched perilla seeds were dissolved in NaOH solution (pH 10.0) at a ratio of 1:10 (*w*/*v*) and stirred for 30, 60, and 90 min. The extract was centrifuged at 6000 rpm for 15 min at 4 °C, and the supernatant was adjusted to pH 4.0 with 1 M HCl. The mixture was centrifuged at 6000 rpm for 15 min at 4 °C, and the resulting precipitate was washed, adjusted pH to 7.0 with 1 M NaOH, lyophilized, and stored at −20 °C. Protein yield was calculated from Equation (1). The condition with the highest Se and protein content will be selected for future experiments with control samples.

(1) $$Protein\;yield\;\left(\%\right)=\left(\frac{gram\;of\;protein\;powder\;obtained}{gram\;of\;Se\;–perilla\;seed\;used}\right)\times 100$$

The enzymatic hydrolysis of Se-enriched perilla seed protein was investigated using two different proteases—Alcalase (Al) and Flavourzyme (Fl)—and combined enzymes (FlAl) [21,22]. For Alcalase hydrolysis, the Se-enriched perilla protein was dissolved in distilled water at a ratio of 1% (*w*/*v*), and the pH of the mixture was adjusted to 8.5 with 1 M NaOH and stirred by a magnetic bar at 55 °C. Alcalase was added to the slurry at an enzyme-to-substrate ratio (E/S) of 2% *w*/*w* for 5 h. The optimal pH conditions were maintained by dropping 1 M NaOH solution. After enzymatic hydrolysis, the reaction mixture was heated at 95 °C for 10 min to inactivate the enzyme before centrifugation. The supernatant containing the target peptides was used as the perilla peptide (PP) and perilla Se-peptide (PseP). For Flavourzyme hydrolysis, the optimal pH and temperature of Flavourzyme was 7.0 and 50 °C, respectively. After adjusting to the optimal condition, Flavourzyme was added to the slurry at an E/S ratio of 2% *w*/*w* for 3 h. Then, we followed the same steps as in Alcalase hydrolysis. Lastly, combined enzymes hydrolysis was investigated. First, Se-enriched perilla seed protein solution at 1% (*w*/*v*) was prepared to the optimal condition for Flavourzyme (pH 7.0 at 50 °C). The enzymatic hydrolysis was initiated using Flavourzyme at an E/S ratio of 2% *w*/*w* for 3 h. Then, the hydrolysate solution was heated to 55 °C as the optimal temperature of Alcalase activity. The secondary hydrolysis stage was started by Alcalase addition at pH 8.5 and an E/S ratio of 2% w-w for 5 h, and then the enzymatic reaction was stopped by heating at the mentioned condition. The PPs and PsePs were lyophilized and stored at −20 °C for the determination of degree of hydrolysis (DH), Se and protein contents, and antioxidant activity. The Se-peptides with the highest DH, Se and protein content, and antioxidant activities were chosen for further purification.

### 2.4. Partial Purification of Se-Peptides

#### 2.4.1. Ultrafiltration

The Se-peptides with the highest degree of hydrolysate were passed through an ultrafiltration (UF) membrane with a molecular weight (MW) cut-off (MWCO) of 3, 5, and 10 kDa using an Amicon stirred UF cell 8050 (Millipore, Billerica, MA, USA) [23]. The permeate from each MW cut-off membrane was separated into peptide fractions of >10, 5–10, 3–5, and <3 kDa at F1, F2, F3, and F4 respectively. The fractions of Se-peptides were lyophilized and kept at −20 °C for later analysis. The fraction with the highest Se content and antioxidant activity were selected for anticancer activity, ACE inhibition activity, and further purification using Prep-HPLC.

#### 2.4.2. Preparative High-Performance Liquid Chromatography (Prep-HPLC)

The Se-peptide fraction with the highest antioxidant activity was further purified using a Versatile Preparative HPLC system LC-forte/R-II (YMC Co., Ltd., Kyoto, Japan), with a Sephadex G-25 gel filtration column (175 mm × 750 mm) and detection wavelength of 220 and 280 nm [24]. The concentration of the sample was 10 mg/mL, and the injection volume was 10 mL. The flow rate was 8 mL/min using HPLC water as a mobile phase. The fraction was collected, then lyophilized. Peptides obtained through size-exclusion chromatography were named SPs, while Se-peptides were referred to as SSePs. The antioxidant activity, anticancer activity, ACE inhibition activity, and selenopeptide sequences were determined.

#### 2.4.3. Selenopeptide Sequences Identification

Peptide sequencing was performed using a slightly modified version of an established protocol [25,26]. Peptides from Se-enriched perilla seeds were extracted by homogenizing the sample in 0.1% formic acid, followed by centrifugation, solid-phase extraction, rotary vacuum evaporation, and lyophilization. The peptides were then quantified and adjusted to a concentration of 0.2 ng/µL for LC-MS/MS analysis. The analysis was carried out using an Orbitrap Exploris 480 system equipped with a C18 reverse-phase column and a 45 min acetonitrile gradient. De novo peptide sequencing was performed using PEAKS Studio X, with spectral data converted through ProteoWizard and interpreted based on b- and y-ion fragmentation patterns.

### 2.5. Determinations of Se-Enriched Perilla frutescens

#### 2.5.1. Protein Analysis

The protein contents of samples were determined using the Kjeldahl method described by AACC [27] and Kamboj et al. [28]. The percentage of nitrogen was calculated and subsequently converted to protein content by multiplying by a nitrogen-to-protein conversion factor of 6.25.

#### 2.5.2. Total Selenium Content

The Se contents of the samples were determined by the method described by Mezeyová [29]. The digestion of samples took place in a microwave digestion oven. Then, 0.15 g of sample was added to a digestive vessel and mixed with 4 mL of concentrated nitric acid (65% HNO_3_) and 2 mL of concentrated hydrogen peroxide (30% H_2_O_2_). The mixtures were digested at 190 °C for 20 min. The digestion product was adjusted to 25 mL in a volumetric flask. Selenium content was determined using the atomic absorption spectroscopy (AAS) technique with the non-flame atomization technique (novAA800, Analytik Jena, Jena, Germany). The wavelength used for Se analysis was 196.0 nanometers. The quantity of Se in the samples was calculated and compared against a standard curve to determine the condition with the highest Se content for the next experiment.

#### 2.5.3. Degree of Hydrolysis (DH)

The degree of hydrolysis (DH) is a critical parameter in evaluating the extent of protein breakdown during enzymatic hydrolysis. In this study, the DH of perilla peptide hydrolyzed by Alcalase, Flavourzyme, and their combination was analyzed at two different conditions: 0 ppm and 80 ppm enzyme concentrations. The degree of hydrolysis of perilla protein hydrolysate was determined using the pH-stat method according to the method described by Zhang et al. [30], with slight modifications. During hydrolysis, peptide bonds broke down into free amino and carboxyl groups, and the dissociation states of the two groups varied under different pH conditions. To maintain the original pH, an NaOH solution was added to the mixture. As a result, the consumption of NaOH solution was proportional to the amount of carboxyl groups formed by the reaction, as well as the hydrolyzed peptide bonds and DH of the hydrolysate. The DH of the hydrolysate was calculated by Equation (2):

(2) $$DH\left(\%\right)=\frac{V\;\times N}{\alpha \;\times {\;M}_{p}\times h_{tot}}\times 100$$

where *V* is the consumed volume of NaOH solution (mL); *N* is the molarity of NaOH solution (mol/L); *M_p_* is the total mass of protein in hydrolyzed sample; *h_tot_* is the total amount of peptide bonds per unit mass of protein (mmol/g) (the *h_tot_* of perilla protein was 8.0); and *α* is the average degree of dissociation of the α-NH_2_ groups released during hydrolysis, expressed as

(3) $$\frac{1}{\alpha }=10^{pK\;–pH}+1$$

where *pH* and *pK* are the values at which proteolysis was conducted.

#### 2.5.4. Antioxidant Activity

##### ABTS Radical Scavenging Activity Assay

The ABTS radical scavenging activity assay was performed as previously described [31,32], with slight adjustments. Briefly, 50 µL of the sample at a concentration of 1 mg/mL was added to 96-well plates; then, we added 200 µL of the ABTS^•+^ solution. The mixture was kept in the dark for 6 min. The absorbance of the solution was measured at 734 nm.

##### Ferric Reducing Antioxidant Power (FRAP) Assay

The FRAP radical scavenging assay was modified from the method reported by Candra et al. [33]. Briefly, the TPTZ solution was mixed with the FeCl_3_•6H_2_O solution and acetate buffer pH 3.6 in a 1:1:10 ratio, resulting in the FRAP reagent for testing the antioxidant capacity of the samples. Then, 180 µL of the prepared FRAP reagent was added to 20 µL of the sample solution at the concentration of 1 mg/mL. The mixture was left for 5 min before measuring the absorbance at 595 nm.

#### 2.5.5. ACE Inhibitory Activity

ACE inhibitory activity was determined using the ACE Kit-WST (Dojindo Laboratories, Kumamoto, Japan), following the manufacturer’s instructions. Briefly, 20 µL of the sample (0.25 mg/mL), working enzyme solution, and substrate buffer were mixed in 96-well plates and incubated at 37 °C for 1 h. Subsequently, 200 µL of the indicator working solution was added. The plates were shaken and incubated at room temperature for 10 min. The absorbance was measured at 450 nm [34]. The ACE inhibitory activity was determined using Equation (4):

(4) $$ACE\;Inhibitory\;Activity\left(\%\right)=\frac{\left(A_{blank1}–A_{sample}\right)}{\left(A_{blank1}–A_{blank2}\right)}\times 100$$

where A_blank 1_ is the absorbance of positive control (no ACE inhibition); A_blank 2_ is the absorbance of reagent blank; and A_sample_ is the absorbance of the reaction mixture.

#### 2.5.6. Cell Viability Assay

The cell viability of Se-peptides was determined using the MTT (3-(4,5-dimethylthiazol-2-yl)-2,5-diphenyltetrazolium bromide) assay, as described by Hankittichai et al. [35], with minor modifications. Se-peptides extracted from Se-enriched *Perilla frutescens* were tested on both normal and cancerous cell lines. The human lung cancer cells (ATCC^®^ CCL-185 ^TM^) were obtained from the American Type Culture Collection (ATCC, Manasssas, VA, USA). Cells were seeded into 96-well plates at a density of 5 × 10^4^ cells/well in complete medium and incubated at 37 °C with 5% CO_2_ for 24 h. After incubation, cells were treated with Se-peptides samples, including the F1 fraction, SP, and SSeP, at concentrations ranging from 0.25 to 1 mg/mL. Vehicle controls (0.001–0.05% DMSO) were also included. Following 48 h of treatment, 10 µL of MTT solution (0.4 mg/mL in PBS, pH 7.4) was added to each well, and the plates were incubated for 30 min at 37 °C in the dark. After removing the supernatant, 200 µL of DMSO was added to the wells to dissolve the formazan crystals. Absorbance was read at 570 nm using microplate readers. The assay was performed in triplicate across three independent experiments (*n* = 9).

### 2.6. Statistical Analysis

All experiments were expressed as means  ±  standard deviation (SD). Analysis of variance (ANOVA) was performed using SPSS statistical software (version 17.0, Chicago, IL, USA). The significance in differences was determined by Duncan’s multiple range test (*p*  <  0.05).

## 3. Results and Discussion

### 3.1. Selenium Biotransformation in Se-Enriched Perilla Seeds

The chemical composition of Se-enriched perilla seeds is shown in Table 1. The total Se content in perilla seeds increased from 1.11 µg/g to 164.62 µg/g with higher Se supplementation. The trace amount of Se detected in the control sample suggests that perilla seeds naturally contain Se [36]. In contrast, the fat content varied between 42.08% and 44.18%, and the protein content ranged from 37.34% to 39.05%, showing minimal changes despite Se treatment. These results indicated that germinating perilla seeds in a selenite solution is an efficient method for Se biofortification of perilla seeds. The accumulation of Se in seeds may be attributed to different mechanisms at various germination stages. At the initial stage, the uptake of selenite is primarily driven by the water potential gradient between seeds and their environment. During imbibition, selenite may enter the seed along with water through the aleurone layer and reach internal cells. As germination progresses, seeds resume metabolic activity, and enzymes catalyze the conversion of selenide into selenocysteine, facilitating the biosynthesis of Se-containing proteins [37]. These findings align with previous studies on other crops. For instance, in alfalfa sprouts, Se content significantly increased with higher Se concentrations, while germination rates and biomass were adversely affected beyond certain thresholds [17]. Similarly, in brown rice, Se accumulation increased with external Se concentration up to 180 µmol/L [38]. Moreover, D’Amato et al. [39] found that increasing Se concentration increased the organic and inorganic Se content of rice sprouts. In the case of perilla seeds, optimal protein content and Se accumulation were observed at Se concentrations of 80 and 100 ppm. However, concentrations exceeding 100 ppm led to reduced germination and seedling development, likely due to Se-induced phytotoxicity by either generating reactive oxygen species or malformed selenoprotein. This toxicity is caused by excessive reactive oxygen species (ROS) generated from selenite or by malformed selenoproteins resulting from the replacement of Cys and Met with Se analogues such as SeCys and SeMet. These substitutions can lead to structural instability, as SeCys forms longer, more reactive disulfide bonds, increasing oxidative stress in plant tissues [40,41,42]. This pattern mirrors observations in purple radish sprouts, where high concentrations of Se had a detrimental effect on seedling growth [43]. Since there was no significant difference between 80 and 100 ppm Se concentrations in terms of Se enrichment and seed viability, 80 ppm was selected as the optimal concentration to produce Se-enriched perilla seeds.

### 3.2. Selenopeptide Preparation and Antioxidant Activities

#### 3.2.1. Effect of Protein Extraction Time on Protein Yield, Protein Content, and Se Content

The Se-enriched perilla protein extracted by the alkaline extraction method is presented in Table 2. The protein yields obtained after 30, 60, and 90 min of extraction were 11.48 ± 0.44%, 11.70 ± 0.10%, and 12.33 ± 0.04%, respectively, showing a slight increase over time. The total protein content ranged from 78.71% to 79.50%, while total Se content decreased from 77.24 to 64.15 µg/g sample with prolonged extraction. The increase in protein yield can be attributed to the ability of alkaline extraction to disrupt hydrogen bonds and disulfide linkages, enhancing protein solubility [44]. At alkaline conditions (pH 8–12), protein exhibits an increased net surface charge, which enhances water solubility by strengthening electrostatic repulsion between molecules. Additionally, proteins are more likely to remain soluble when interactions with the solvent dominate protein–protein interactions [45,46]. On the other hand, Se content declined over time, likely due to the instability of SeCys under alkaline pH. SeCys can degrade into dehydroalanine, releasing Se as selenide or selenate/selenite. Additionally, protein unfolding may expose SeCys to oxidation and volatilization, and Se may precipitate or chelate with metal ions, further the availability of Se in the extracted fraction [47,48]. Moreover, the decrease in total Se could be because of the loss of volatile Se compounds, such as dimethyl selenide (DMSe) and dimethyl diselenide (DmeDSe) [49]. Since the total Se content of protein from 30 min extraction was the highest, this condition was selected for protein extraction.

#### 3.2.2. Effects of Enzymatic Hydrolysates on DH and Se Content of Se-Peptide

**Figure 1:**
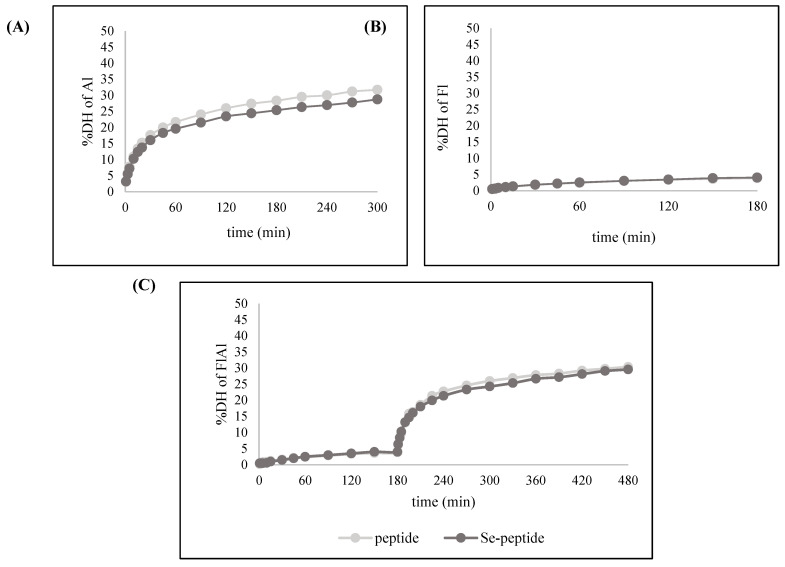
The hydrolysis curves of different protease hydrolysates ((**A**): DH of Alcalase hydrolysate; (**B**): DH of Flavourzyme; (**C**): DH of combined enzymes; DH refers to degree of hydrolysate).

The Se-enriched perilla seed peptide from Se-containing germinated perilla seed, obtained using different proteases, is presented in Table 3. The protein contents of perilla peptides (PPs) derived from all enzymatic hydrolysates ranged from 61.20 to 68.08%, with no significant difference observed (*p* > 0.05). In contrast, the protein contents of perilla Se-peptides (PsePs) varied significantly depending on the enzyme used (*p* < 0.05), with values of 68.79 ± 0.31% for Fl, 62.79 ± 0.74% for Al, and 59.22 ± 1.25% for FlAl. Flavourzyme hydrolysates exhibited the highest Se content (1.69 ± 0.10 µg/g in PPs and 97.17 ± 0.69 µg/g in PsePs), while Alcalase and combination enzyme hydrolysates showed lower Se contents (1.50 ± 0.08 µg/g in PPs and 89.30 ± 0.74 µg/g in PsePs; 1.53 ± 0.06 µg/g in PPs and 84.60 ± 0.68 µg/g in PsePs, respectively), with significant differences (*p* < 0.05). The degree of hydrolysis (DH) values for the different enzymatic treatments (Figure 1) were as follows: Al showed DH values of 31.73 ± 0.39% in PPs and 28.75 ± 0.26% in PsePs. Fl resulted in significantly lower DH values of 3.87% in PPs and 4.04% in PsePs. The combination of Flavourzyme and Alcalase (FlAl) produced DH values of 30.39% in PPs and 29.58% in PsePs, comparable to those obtained with Alcalase alone. The results suggest a slight inhibition of hydrolysis by Se at higher concentrations in Al. The lower DH values observed with Fl are likely due to its exopeptidase activity, which limits extensive peptide bond cleavage. The similar DH values of Alcalase alone and the combined treatment indicate that Alcalase played the dominant role in hydrolysis. This may be attributed to its broad specificity, particularly its ability to cleave peptide bonds in serine (Ser) residue, which are abundant in perilla seed protein [5]. Similar results were observed in Se-enriched brown rice peptides, which, when hydrolyzed by Alcalase, exhibited the highest DH compared with Neutrase, flavorase, and papain [12]. In addition to serine, Alcalase can also cleave at Se-chelating amino acids, including Glu, Met, and Lys residues, when these amino acids are located the P_1_ position [12,50]. This broader substrate specificity contributes to the higher DH observed with Alcalase compared with Flavourzyme. Additionally, the DH of Se-enriched perilla seed peptides increased rapidly during the first 60 min immediately after adding Alcalase to the mixture, followed by a slower rise. This suggested that during the initial hour, the enzymes had maximum interaction with the protein, but as the substrate gradually decreased, the hydrolysis rate declined. Similar trends were observed in previous studies on the hydrolysis of pecan meals and wheat germ protein [51,52].

#### 3.2.3. Antioxidant Activity of Se-Peptides

The antioxidant activities of Se-enriched perilla peptides derived from enzymatic hydrolysates were performed using ABTS and FRAP assays, as shown in Table 3. For ABTS activity, Al exhibited high radical scavenging capacity, with inhibition activity at 80.28 ± 1.05% in PPs and 79.14 ± 0.64% in PSePs, while Fl had lower activity at 68.77 ± 0.35% and 68.06 ± 1.00%, respectively. FlAl showed the highest inhibition (80.50 ± 1.70% in PPs, 79.42 ± 0.50% in PSePs), with no significant difference between PPs and PSePs (*p* > 0.05). Samples with higher DH (e.g., Al, and FlAl) showed greater ABTS activity, likely due to an increased release of functional amino acids such as Try and Cys, which contribute to radical scavenging through H-atom transfer (HAT) and resonance stabilization [53]. These results align with previous research that Alcalase hydrolysates exhibited higher potency than Flavourzyme hydrolysates, highlighting the role of enzyme specificity in modulating ABTS activity [14,15]. In the FRAP assay, Fl demonstrated the highest reducing power, with an inhibition value of 70.34 ± 1.40% in PPs and a significantly lower inhibition of 64.86 ± 2.08% in PSePs (*p* < 0.05). FlAl and Al treatments showed moderate activity, with no significant difference between PPs and PSePs for Al (*p* > 0.05). In PSePs, the FRAP activity of FlAl was not significantly different from that of Al alone (*p* > 0.05), indicating that Fl pre-treatment did not enhance reducing capacity [54]. This was supported by Liu et al. [55], who highlighted that Cys analogues containing the selenol (-SeH) group possess strong reducing power due to their electron-donating capabilities. Considering Se content, DH, and antioxidant activity, hydrolysis with Alcalase for 300 min (which yielded ~30% DH) and high Se content were selected for ultrafiltration membrane fractionation for further analysis.

### 3.3. Membrane Ultrafiltration of Se-Peptides

Se-peptide samples were fractionated using ultrafiltration membranes with different molecular-weight cut-offs (MWCO: 10, 5, and 3 kDa) to determine the distribution of Se across molecular size ranges. This approach yielded four fractions: F1 (>10 kDa), F2 (5–10 kDa), F3 (3–5 kDa), and F4 (<3 kDa) (Table 4). The results showed that Se was distributed across all fractions, with the highest Se content detected in the F1 (>10 kDa) fraction (124.58 ± 6.74 µg/g sample). In contrast, PPs exhibited much lower Se content (2.39–2.72 µg/g sample), indicating that external inorganic Se was successfully converted into peptide-bound forms and accumulated in various molecular-weight proteins. These findings were in line with previous research showing that Se can bind to and accumulate in protein structures of different sizes during biofortification [9,56]. The preferential accumulation of Se in higher-molecular-weight fractions suggests that larger peptide complexes may provide more binding sites or structural compatibility for Se incorporation. Liu et al. [37] reported a similar range of MW Se-enriched brown rice proteins, which ranged from 13.6 to 121.4 kDa. This distribution pattern also supports the notion that Se is integrated during or following peptide synthesis, likely through binding with Se-chelating amino acids or the incorporation of Ses like SeCys and SeMet [12].

#### 3.3.1. Antioxidant Activities of Se-Peptides Fractions

The antioxidant potential of bioactive peptide fractions with varying MWs was evaluated using ABTS and FRAP assay, as shown in Table 4. High-MW fractions (F1) exhibited the strongest ABTS scavenging activity, with values of 89.88 ± 0.10% in PPs and 83.63 ± 2.24% in PSePs, indicating that larger peptides may contain a greater number of functional groups capable of engaging in hydrogen atom-transfer reactions, thereby enhancing their radical neutralization. Phongthai et al. [57] reported that both hydrophobic residues (e.g., Met, Phe, Val) and hydrophilic residues (e.g., Lys, His, Arg) contribute to ABTS scavenging, with Phe donating protons through its benzene ring. Mid-sized peptides (F2) showed ABTS scavenging activity of 78.68 ± 2.05% in PPs, which slightly increased to 81.67 ± 1.30% in PSePs, while lower-MW fractions (F3, F4) exhibited lower scavenging activity, indicating that peptide size and structure are crucial for effective radical scavenging [14]. The trend observed here contrasts with previous reports suggesting stronger ABTS activity in <3 kDa fractions [14,20], possibly due to the influence of Se, which may alter electronic characteristics or conformational stability. A similar pattern was observed in FRAP activity, where the F1 fraction showed the highest reducing power (60.65 ± 1.33% in PPs and 59.59 ± 1.06% in PSePs), while the F4 fraction showed the lowest activity (36.57 ± 0.81% in PPs, slightly increasing to 38.39 ± 1.29% in PSePs). This result supports prior findings that larger peptides may possess more electron-donating residues, enhancing their ability to reduce ferric ions [58].

#### 3.3.2. ACE Inhibitory and Anticancer Activities of Se-Peptides Fractions

**Figure 2:**
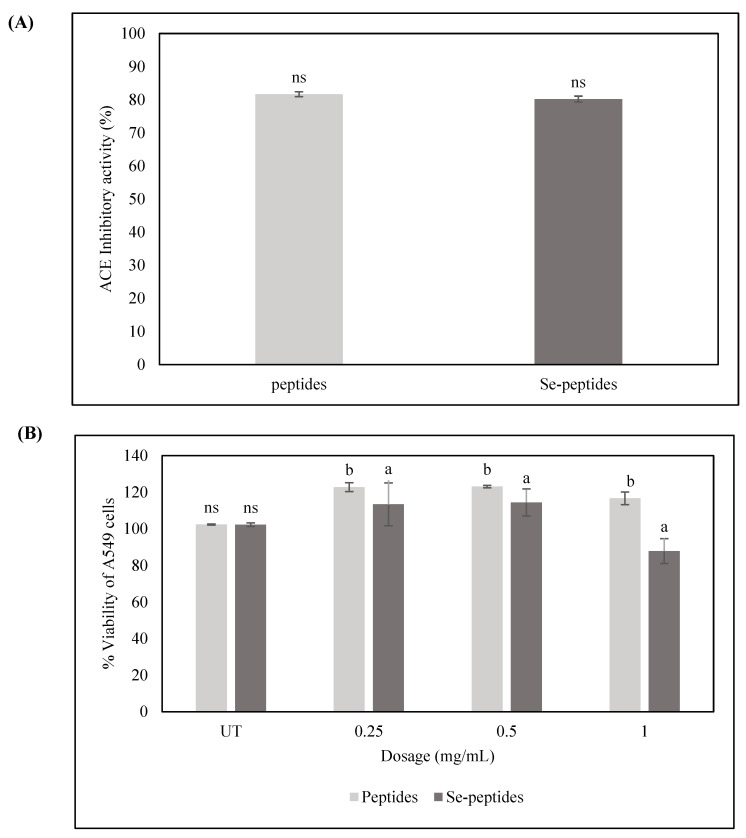
The ACE inhibitory and anticancer activities of Se-peptides ((**A**): ACE inhibitory activity; (**B**): % viability of A549 cells). Lowercase letters (a and b) correspond to the difference between samples in the same concentration; ns refers to not significant; UT, untreated.

The ACE inhibitory activity of the F1 fraction from both peptides and Se-peptides is presented in Figure 2A. Both peptides and Se-peptides exhibited strong ACE inhibitory activity, with values of 81.65 ± 0.73% and 80.19 ± 0.89%, respectively, with no significant differences (*p* > 0.05). Previous studies have indicated that the amino acid composition, particularly the presence of hydrophobic residues, significantly influences ACE inhibition, as ACE preferentially interacts with peptides rich in these amino acids. Higher-MW peptides are more likely to contain such hydrophobic residues, which may explain their elevated inhibitory activity [59]. This result is in line with the work of Mansinhbhai et al. [60], who demonstrated that the >10 kDa fraction of whey protein concentrate hydrolyzed by Alcalase showed the highest ACE inhibitory activity, reinforcing the idea that molecular size plays a key role in ACE inhibition.

The effect of peptides and Se-peptides derived from *Perilla frutescens* peptides on the viability of A549 human lung cancer cells was evaluated using an MTT assay (Figure 2B). Se-peptides at a concentration of 1 mg/mL exhibited minimal cytotoxicity, with cell viability remaining at 87.82 ± 6.75%, indicating a slight inhibitory effect on cancer cell growth. In contrast, non-selenized peptides significantly increased A549 cell viability to 116.67 ± 3.45%, suggesting a potential proliferative effect. This aligns with Zhang et al. [61], who reported that Se-containing peptides modulate redox balance and activate the Nrf2 pathway, which may suppress cancer cell growth. These findings highlight the impact of Se incorporation on peptide bioactivity. Owing to the notable bioactivities of the F1 peptides, this fraction was selected for further purification using size-exclusion chromatography and subsequent biological activity analysis.

### 3.4. Purification, Antioxidants, ACE Inhibition, and Anticancer Activities of Se-Peptide Using Size-Exclusion Chromatography

The >10 kDa Se-peptide fraction was separated using a Sephadex G-25 gel filtration column via Prep-HPLC. As shown in Figure S1, the elution profile indicates that the peptides obtained were relatively pure. The antioxidant activity of Se-peptides purified using size-exclusion chromatography is shown in Table 5. The ABTS assay results show a scavenging activity of 63.62 ± 0.85% in peptides and 66.30 ± 0.91% in Se-peptides. This antioxidant potential might be attributed to the presence of amino acids such as Cys, Lys, His, Arg, Val, Phe, and Met in the sequences, which have been reported to exhibit ABTS radical scavenging activity [55]. Specifically, cysteine contains the -SeH group, which has strong electron-donating and hydrogen-donating capabilities, contributing to its high antioxidant activity. Methionine also shows free radical scavenging ability due to its susceptibility to oxidation into methionine selenoxide, and cysteine may support this process by donating Se-bound hydrogen. Moreover, the presence of Phe and Lys could contribute to radical scavenging via electron donation from the benzene ring and the -NH_2_ group, respectively [55,62]. The results of the FRAP assay revealed no significant difference (*p* > 0.05) in ferric-reducing antioxidant capacity between the SP and SSeP (56.30 ± 0.33% and 54.93 ± 0.96%, respectively). This may be due to the FRAP assay primarily measuring single-electron transfer from electron-rich amino acids (e.g., Tyr, Trp, Cys), which was not presented in the peptide sequences from perilla seed peptides.

The SSeP exhibited significantly higher ACE inhibitory activity (83.87 ± 0.75%) compared to the SP, which showed 74.30 ± 1.19% inhibition (*p* < 0.05) in Table 5. This increased ACE inhibitory activity may be linked to the presence of bioactive peptides containing hydrophobic (Leu, Val), acidic (Glu, Asp), and Se-containing residues, which are known to enhance enzyme inhibition. According to Olalere et al. [59], peptides with Phe, Leu, or Arg at the N1 position, along with other specific amino acids at N2, N3, C1, C2, and C3, demonstrate notable ACE inhibitory potential. Additionally, Se incorporation could further improve this activity by influencing peptide structure and stability. Moreover, SSePs showed slightly enhanced anticancer activity against A549 human lung cancer cells. The results indicated that at the treatment concentration of 1 mg/mL, SSePs showed significantly lower cell viability compared to SPs (88.95 ± 1.36% in SPs, and 85.88 ± 3.72% in SSePs). This effect might be attributed to the presence of Pro and Trp residues, which are known to promote structural stability and facilitate cell membrane interaction, respectively. Additionally, Gly and Val may support solubility and interaction with intracellular targets, further contributing to the observed cytotoxicity [6,63]. The Se-peptides from *Perilla frutescens* showed potential in antioxidant production, ACE inhibition, and anticancer activities.

### 3.5. Identification of Amino Acid Sequence in Se-Peptides from Se-Enriched Perilla frutescens

The PSeP with a MW >10 kDa purified using size-exclusion chromatography was then analyzed for its amino acid sequence using the LC-MS/MS spectrum method (Figure S2). It was found that the mass distribution histogram of perilla peptides and Se-enriched perilla peptides was in the range of 1000–2200 Da, with de novo scores ranging from 80% to 93% (Table 6). The sequences identified in this study were predominantly long chains (exceeding 10 amino acids) including sulfur containing amino acids such as Cys and Met. The sequences also contain Se-chelating amino acids such as Glu, Arg, Asp, and Lys. This may explain the higher Se content in Se-enriched *Perilla frutescens* compared to the non-enriched counterpart. Se-peptides exhibit antioxidant activity through multiple mechanisms, including scavenging double-electron oxidants, enhancing oxidative damage repair, binding metal ions, and functioning as catalytic residues in various protective enzymes. The presence of hydrophobic (Cys, Met, Lys, His, Pro, Leu) and aromatic amino acids (Ser) in the Se-peptides likely contributed to their strong antioxidant activity by enhancing radical scavenging and interaction with lipid membranes, which facilitates access to hydrophobic radical species. Moreover, basic amino acids such as Arg within the peptide sequences supported metal ion-chelating activity [12,64]. ACE inhibitory activity may be influenced by amino acid positioning. The C-terminals (C1, C2, and C3) are more predominant in ACE binding. Six out of nine sequences had Phe, Leu, and Arg on the N1 terminal, Pro on the N2, C1, C2, and C3 terminals, and Arg on the C2 terminal, which has been reported to exhibit ACE inhibitory activity [59]. Furthermore, the inclusion of Pro, Trp, Ser, Gly, and Val in the peptide sequences may enhance their biological activity by affecting solubility, structural stability, and interactions with specific target molecules [6]. These findings suggest that Se-enriched *Perilla frutescens* seeds are a promising source of bioactive Se-peptides.

## 4. Conclusions

The Se biotransformation of *Perilla frutescens* presents an effective strategy for producing bioactive Se-peptides with notable antioxidants, ACE inhibition, and anticancer activities. Se-peptides hydrolyzed by Alcalase led to a higher degree of hydrolysis and strong antioxidant activities. Further fractionation revealed that high-molecular-weight Se-peptides (>10 kDa) contained the highest Se content, exhibited the highest antioxidant activities, and had high potential in ACE inhibition and anticancer effects. LC-MS/MS analysis confirmed that selenopeptides from Se-enriched perilla seeds contain selenoamino acids and Se-chelating amino acids, which enhance ACE inhibition and anticancer activities. In conclusion, Se-enriched perilla peptides have strong potential as a functional food and as nutraceutical ingredients. Further research will help clarify their applications and health benefits, reinforcing the value of perilla seeds as a promising functional food source. However, the application of Se-peptides in the food and pharmaceutical industries requires further investigation, particularly regarding their bioavailability, in vivo bioactivity, and toxicity, including critical trials in humans. Future studies should focus on their metabolic pathways, assessing bioavailability in vivo, and developing advanced delivery systems, such as nanoencapsulation, to enhance stability and targeted release in food applications.

## Figures and Tables

**Table 1:** Effect of Se concentrations on fat, protein, and Se content of perilla seeds.

Se Concentration (ppm)	Fat Content ^ns^ (%)	Protein Content (%)	Se Content (µg/g Sample)
0 (control)	44.18 ± 0.63	37.34 ± 0.29 ^b^	1.11 ± 0.02 ^e^
20	43.97 ± 0.25	37.52 ± 0.02 ^b^	25.22 ± 0.42 ^d^
40	44.14 ± 1.26	37.72 ± 0.34 ^ab^	68.83 ± 0.74 ^c^
60	42.08 ± 0.94	38.15 ± 0.23 ^ab^	86.64 ± 0.87 ^b^
80	42.87 ± 0.21	39.05 ± 1.14 ^a^	164.07 ± 2.51 ^a^
100	44.02 ± 0.63	38.93 ± 0.10 ^a^	164.62 ± 4.80 ^a^

Data are expressed as means ± SD. Lowercase letters (a, b, c, d, e) correspond to the difference between sample in the same column (*p* < 0.05). ns correspond to non-significant difference between sample (*p* > 0.05).

**Table 2:** Effect of extraction time on protein yield, protein content, and Se content of selenoprotein.

Extraction Time (min)	Yield (%)	Protein Content (%)	Se Content (µg/g Sample)
30	11.48 ± 0.44 ^b^	78.71 ± 0.00 ^b^	77.24 ± 5.06 ^a^
60	11.70 ± 0.10 ^ab^	79.50 ± 0.15 ^a^	66.67 ± 1.88 ^b^
90	12.33 ± 0.04 ^a^	79.37 ± 0.24 ^a^	64.15 ± 4.94 ^b^

Data are expressed as means ± SD. Lowercase letters (a, b) correspond to the difference between samples in the same column (*p* < 0.05).

**Table 3:** The protein and Se contents of different protease hydrolysates.

Enzymatic Hydrolysates	PP			PSeP		
	Al	Fl	FlAl	Al	Fl	FlAl
**Protein** (**%**)	61.20 ± 5.70 ^ns^	68.08 ± 0.76 ^ns^	65.04 ± 0.92 ^ns^	62.79 ± 0.74 ^B^	68.79 ± 0.31 ^A^	59.22 ± 1.25 ^C^
**Se contents **(**µg/g sample**)	1.50 ± 0.08 ^b^	1.69 ± 0.10 ^a^	1.53 ± 0.06 ^ab^	89.30 ± 0.74 ^B^	97.17 ± 0.69 ^A^	84.60 ± 0.68 ^C^
**DH **(**%**)	31.73 ± 0.39 ^a^	3.87 ± 0.00 ^c^	30.39 ± 0.00 ^b^	28.75 ± 0.26 ^B^	4.04 ± 0.00 ^C^	29.58 ± 0.01 ^A^
**% inhibition of ABTS**	80.28 ± 1.05 ^a^	68.77 ± 0.35 ^b^	80.50 ± 1.70 ^a^	79.14 ± 0.64 ^A^	68.06 ± 1.00 ^B^	79.42 ± 0.50 ^A^
**% inhibition of FRAP**	54.05 ± 0.80 ^c^	70.34 ± 1.41 ^a^	57.30 ± 0.57 ^b^	52.97 ± 1.57 ^B^	64.86 ± 2.08 ^A^	52.12 ± 0.72 ^B^

Data are expressed as means ± SD. (PPs, perilla peptides; PSePs, perilla Se-peptides; DH, degree of hydrolysate; Al, Se-enriched perilla protein Alcalase hydrolysates; Fl, Se-enriched perilla protein Flavourzyme hydrolysates; FlAl, Se-enriched perilla protein Flavourzyme and Alcalase hydrolysates). Lowercase letters (a, b, c) correspond to the difference between samples in the same roll (*p* < 0.05); uppercase letters (A, B, C) correspond to the difference between samples in the same roll (*p* < 0.05); ns refers to not significant.

**Table 4:** Se contents and biological activities of perilla peptides and Se-peptides fractions.

	Fractions	Se Contents (µg/g Sample)	%Inhibition of ABTS	%Inhibition of FRAP
Peptides	F1 (>10 kDa)	2.61 ± 0.52 ^ns^	89.88 ± 0.10 ^a^	60.65 ± 1.33 ^a^
	F2 (5–10 kDa)	2.59 ± 0.09 ^ns^	78.68 ± 2.05 ^b^	51.83 ± 0.85 ^b^
	F3 (3–5 kDa)	2.39 ± 0.17 ^ns^	74.63 ± 1.77 ^c^	40.24 ± 0.62 ^c^
	F4 (<3 kDa)	2.72 ± 0.00 ^ns^	76.71 ± 1.28 ^bc^	36.57 ± 0.81 ^d^
Se-peptides	F1 (>10 kDa)	124.58 ± 6.74 ^A^	83.63 ± 2.24 ^A^	59.59 ± 1.06 ^A^
	F2 (5–10 kDa)	76.48 ± 2.04 ^B^	81.67 ± 1.30 ^A^	53.98 ± 1.60 ^B^
	F3 (3–5 kDa)	60.33 ± 2.16 ^C^	75.19 ± 0.17 ^B^	39.92 ± 1.04 ^C^
	F4 (<3 kDa)	75.86 ± 8.00 ^B^	68.25 ± 0.29 ^C^	38.39 ± 1.29 ^C^

Data are expressed as means ± SD. Lowercase letters (a, b, c, d) correspond to the difference between samples in the same column (*p* < 0.05); uppercase letters (A, B, C) correspond to the difference between samples in the same column (*p* < 0.05); ns refers to not significant.

**Table 5:** Biological activities of Se-peptides after size-exclusion chromatography.

			SP	SSeP
**% Inhibition**	**ABTS**		63.62 ± 0.85 ^b^	66.30 ± 0.91 ^a^
	**FRAP**		56.30 ± 0.33 ^ns^	54.93 ± 0.96 ^ns^
	**ACE**		74.30 ± 1.19 ^b^	83.87 ± 0.75 ^a^
**%viability**	**A549 cells**	UT	100.06 ± 0.93 ^ns^	101.72 ± 2.67 ^ns^
		0.25 mg/mL	95.71 ± 0.12 ^b^	89.33 ± 0.23 ^a^
		0.5 mg/mL	90.82 ± 0.12 ^b^	87.77 ± 1.04 ^a^
		1 mg/mL	88.95 ± 1.36 ^b^	85.88 ± 3.72 ^a^

Data are expressed as means ± SD. (SPs, perilla peptides after size-exclusion; SSePs, perilla Se-peptides after size-exclusion; UT, untreated) Lowercase letters (a and b), correspond to the difference between samples in the same roll; ns refers to not significant.

**Table 6:** Identification of amino acid sequence in Se-peptides from perilla seeds by mass (Da) and de novo score (%).

Sample	Peptide	Amino Acid Sequences	Mass (Da)	*m*/*z*	De Novo Score (%)
peptides	FPPEEMEACL	Phe-Pro-Pro-Glu-Glu-Met-Glu-Ala-Cys-Leu	1164.48	583.26	93
	RMVLPEETEEEEEERS	Arg-Met-Val-Leu-Pro-Glu-Glu-Thr-Glu-Glu-Glu-Glu-Glu-Glu-Arg-Ser	1990.88	996.45	91
	RMVLPEETEEEEERESR	Arg-Met-Val-Leu-Pro-Glu-Glu-Thr-Glu-Glu-Glu-Glu-Glu-Arg-Glu-Ser-Arg	2146.98	716.67	91
	LAGGREDMPPQ	Leu-Ala-Gly-Gly-Arg-Glu-Asp-Met-Pro-Pro-Gln	1169.55	585.78	86
	QGKEDDRGLMVR	Gln-Gly-Lys-Glu-Asp-Asp-Arg-Gly-Leu-Met-Val-Arg	1402.70	468.57	86
	GKEDDRGMLVR	Gly-Lys-Glu-Asp-Asp-Arg-Gly-Met-Leu-Val-Arg	1274.64	425.89	83
	MDLPEERTEEEEESER	Met-Asp-Leu-Pro-Glu-Glu-Arg-Thr-Glu-Glu-Glu-Glu-Glu-Ser-Glu-Arg	2006.84	1004.44	82
	CHEEEERRE	Cys-His-Glu-Glu-Glu-Glu-Arg-Arg-Glu	1215.49	608.76	80
	VMPEETESFEPEPP	Val-Met-Pro-Glu-Glu-Thr-Glu-Ser-Phe-Glu-Pro-Glu-Pro-Pro	1616.69	809.36	80
Se-peptides	RMVLPEETEEEEEERS	Arg-Met-Val-Leu-Pro-Glu-Glu-Thr-Glu-Glu-Glu-Glu-Glu-Glu-Arg-Ser	1990.88	996.45	93
	FPPEEMEACL	Phe-Pro-Pro-Glu-Glu-Met-Glu-Ala-Cys-Leu	1164.48	583.26	91
	LAGGREDMPPQ	Leu-Ala-Gly-Gly-Arg-Glu-Asp-Met-Pro-Pro-Gln	1169.55	585.78	89
	MDLPEERTEEEEESER	Met-Asp-Leu-Pro-Glu-Glu-Arg-Thr-Glu-Glu-Glu-Glu-Glu-Ser-Glu-Arg	2006.84	1004.44	87
	RMVLPEETEEEEERESR	Arg-Met-Val-Leu-Pro-Glu-Glu-Thr-Glu-Glu-Glu-Glu-Glu-Arg-Glu-Ser-Arg	2146.98	716.67	87
	VMPEETESFEPEPP	Val-Met-Pro-Glu-Glu-Thr-Glu-Ser-Phe-Glu-Pro-Glu-Pro-Pro	1616.69	809.36	85
	GKEDDRGMLVR	Gly-Lys-Glu-Asp-Asp-Arg-Gly-Met-Leu-Val-Arg	1274.64	425.89	84
	QGKEDDRGLMVR	Gln-Gly-Lys-Glu-Asp-Asp-Arg-Gly-Leu-Met-Val-Arg	1402.70	468.57	84
	CHEEEERRE	Cys-His-Glu-Glu-Glu-Glu-Arg-Arg-Glu	1215.49	608.76	83

Red color corresponds to amino acids associated with selenium.

## Data Availability

The original contributions presented in this study are included in the article/supplementary material. Further inquiries can be directed to the corresponding author.

## Supplementary Materials

The following supporting information can be downloaded at https://www.mdpi.com/article/10.3390/foods14172988/s1, Figure S1: Elution profile of perilla peptides separated by Prep-HPLC. (**A**) Elution profile of perilla peptides; (**B**) elution profile of perilla Se-peptides; Figure S2: MS/MS spectrum of bioactive peptides and selenopeptides from Se-enriched perilla seeds.

## Abbreviations

Al	Alcalase
Fl	Flavourzyme
AlFl	Combined enzymes
PPs	Perilla peptides
PSePs	Perilla Se-peptides
SPs	Perilla peptides after size exclusion
SSePs	Perilla Se-peptides after size exclusion
