# Supplementary material for: Selenium Biotransformation and Fractionation of Selenopeptide from Germinated Perilla (Perilla frutescens) Seeds
Source: Foods. 2025 Aug 27;14(17):2988. doi: 10.3390/foods14172988 (PMC12428341; doi:10.3390/foods14172988)
Supplement: Supplementary file 1 [file foods-14-02988-s001.zip › Foods-Supplementary Information.pdf]

Supplementary Information

(A)

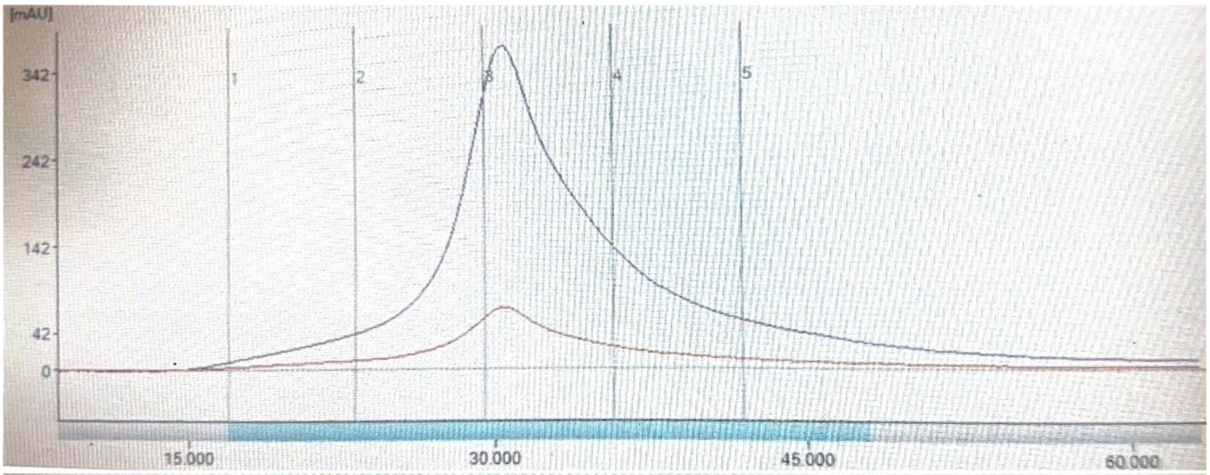

(B)

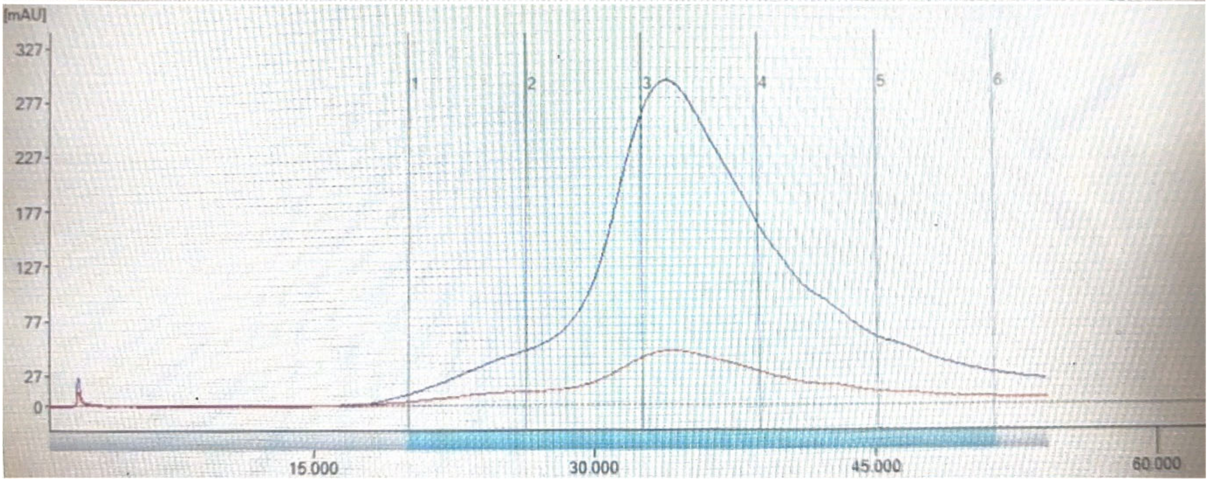

Fig. S1 Elution profile of perilla peptides separated by Prep-HPLC. (A) Elution profile of perilla peptides; (B) elution profile of perilla Se-peptides.

Peptides

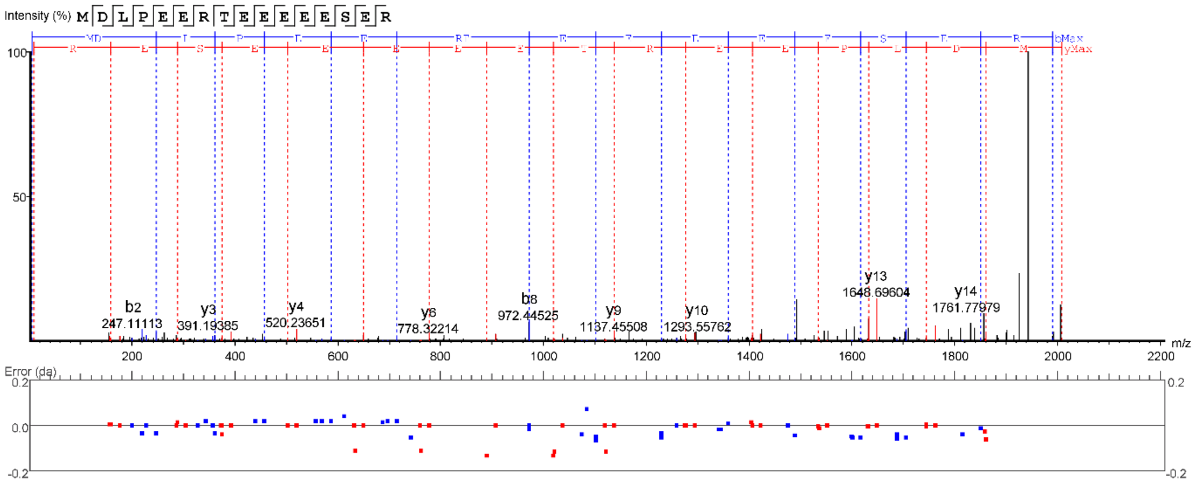
Intensity (%) MDLPEERTEEEEESER
b2 247.11113
y3 391.19385
y4 520.23651
y6 778.32214
b8 972.44525
y9 1137.45508
y10 1293.55762
y13 1648.69604
y14 1761.77979
m/z
Error (da)

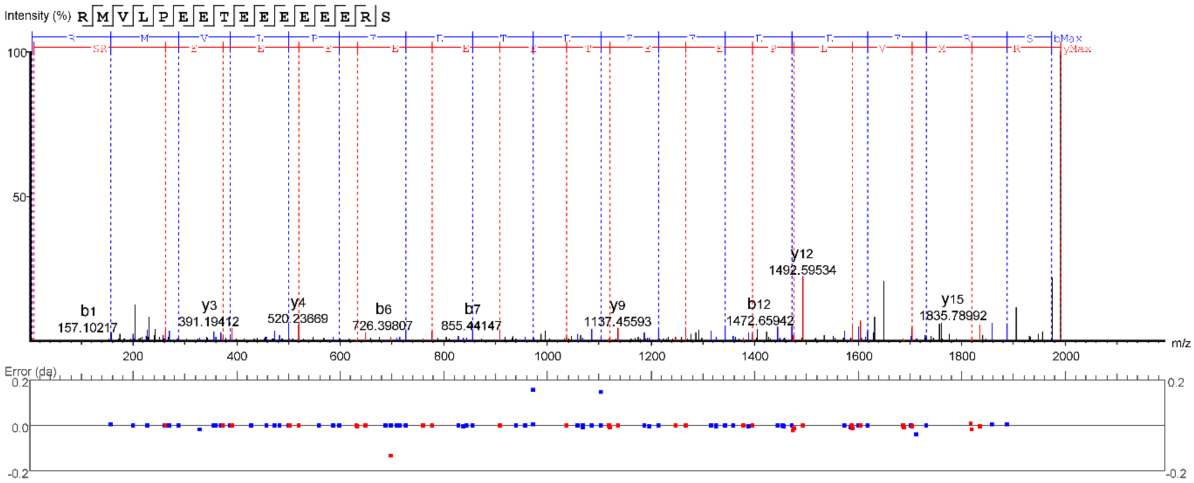
Intensity (%) RMVLPEETEEEEEERS
b1 157.10217
y3 391.19412
y4 520.23669
b6 726.39807
b7 855.44147
y9 1137.45593
b12 1472.65942
y12 1492.59534
y15 1835.78992
m/z
Error (da)

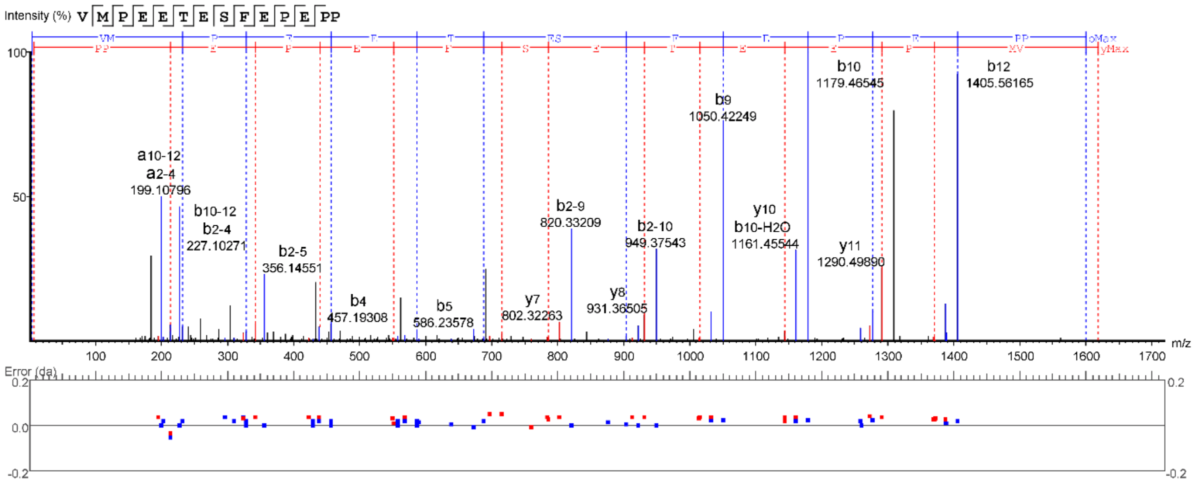
Intensity (%) VMPEETESFEPEPP
a10-12
a2-4
199.10796
b10-12
b2-4
227.10271
b2-5
356.14551
b4
457.19308
b5
586.23578
y7
802.32263
b2-9
820.33209
y8
931.36505
b2-10
949.37543
b9
1050.42249
y10
b10-H2O
1161.45544
b10
1179.46545
y11
1290.49890
b12
1405.56165
m/z
Error (da)

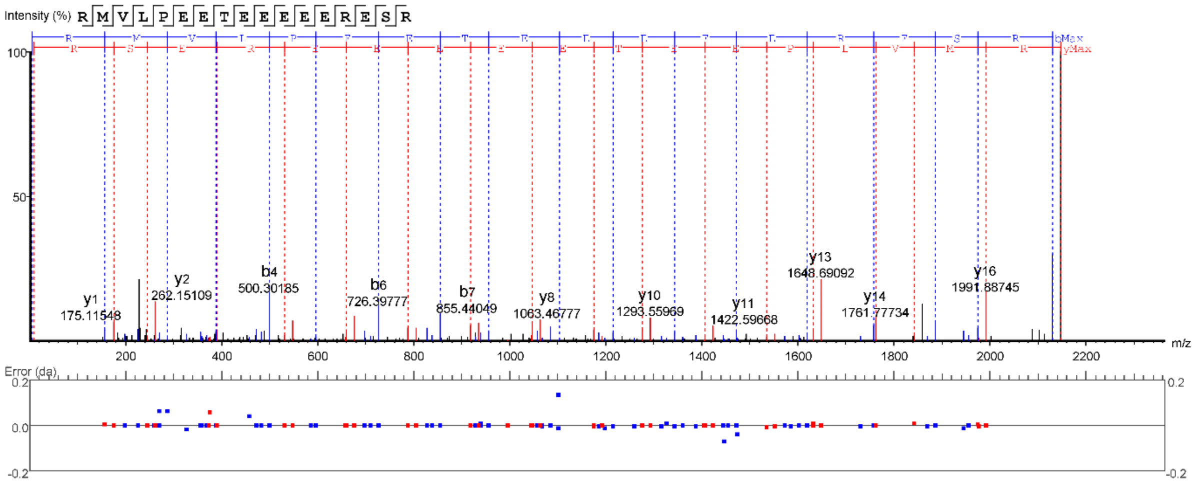
Intensity (%)
RMVLPEETEEEEERESR
y1 175.11548
y2 262.15109
b4 500.30185
b6 726.39777
b7 855.44049
y8 1063.46777
y10 1293.55969
y11 1422.59668
y13 1648.69092
y14 1761.77734
y16 1991.88745
m/z
Error (da)

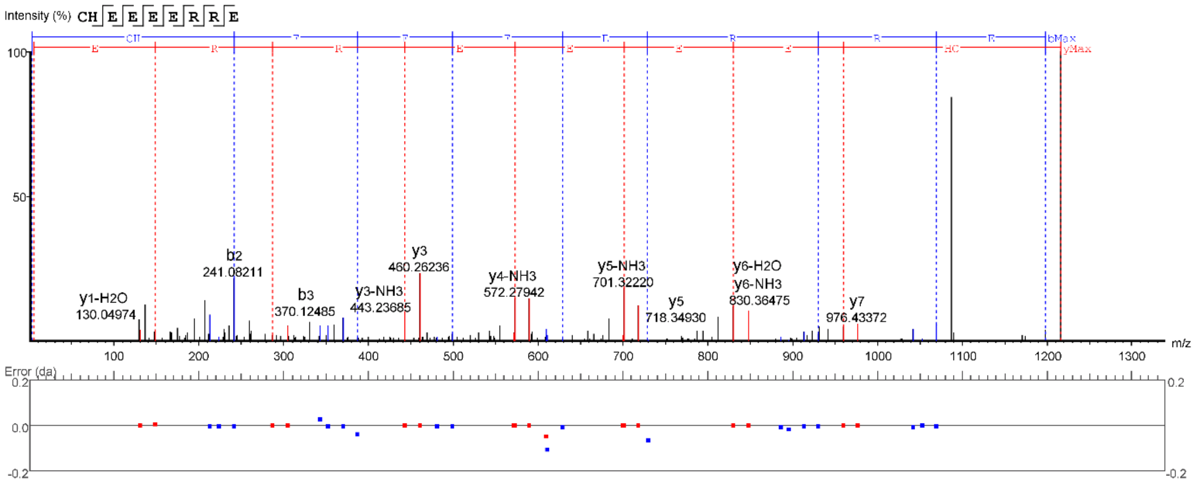
Intensity (%)
CHEEEERRE
y1-H2O 130.04974
b2 241.08211
b3 370.12485
y3-NH3 443.23685
y3 460.26236
y4-NH3 572.27942
y5-NH3 701.32220
y5 718.34930
y6-H2O
y6-NH3 830.36475
y7 976.43372
m/z
Error (da)

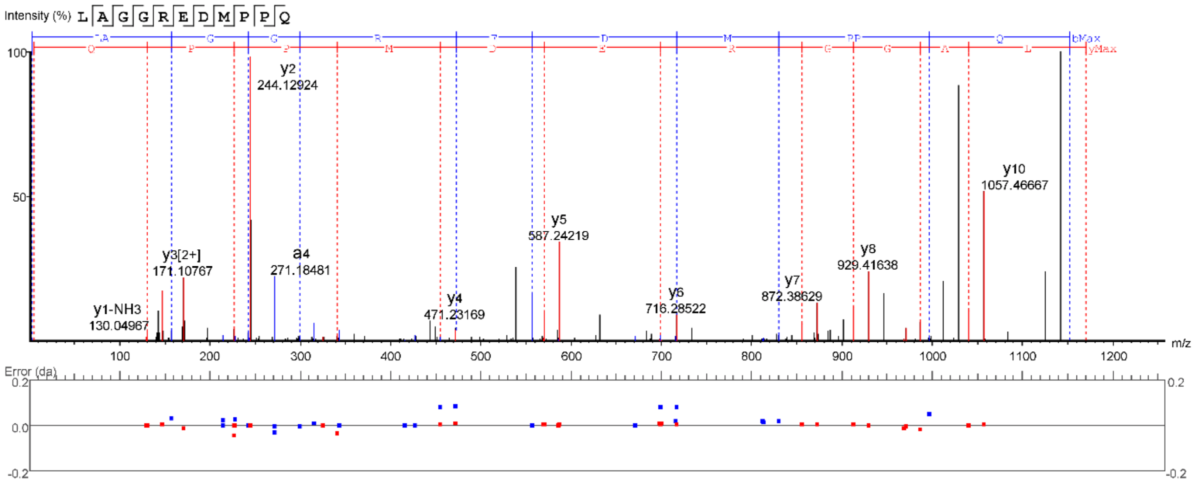
Intensity (%)
LAGGREDMPPQ
y1-NH3 130.04967
y3[2+] 171.10767
y2 244.12924
a4 271.18481
y4 471.23169
y5 587.24219
y6 716.28522
y7 872.38629
y8 929.41638
y10 1057.46667
m/z
Error (da)

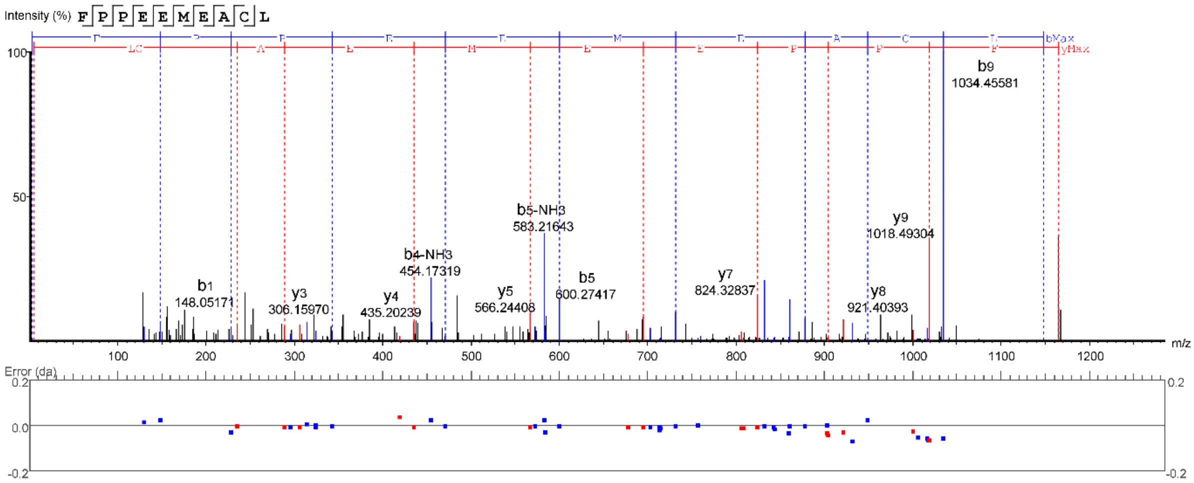

Intensity (%) F P P E E M E A C L
b9
1034.45581
y9
1018.49304
b5-NH3
583.21643
b4-NH3
454.17319
y7
824.32837
b5
600.27417
y5
566.24408
y8
921.40393
b1
148.05171
y3
306.15970
y4
435.20239
m/z
Error (da)

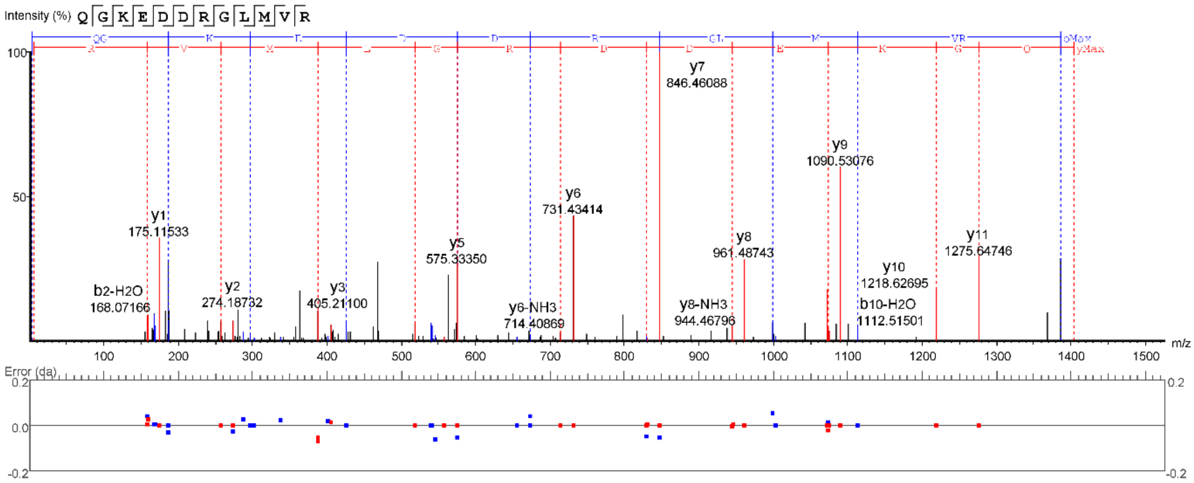

Intensity (%) Q G K E D D R G L M V R
y7
846.46088
y9
1090.53076
y6
731.43414
y1
175.11533
y5
575.33350
y8
961.48743
y11
1275.64746
y10
1218.62695
b2-H2O
168.07166
y2
274.18732
y3
405.21100
y6-NH3
714.40869
y8-NH3
944.46796
b10-H2O
1112.51501
m/z
Error (da)

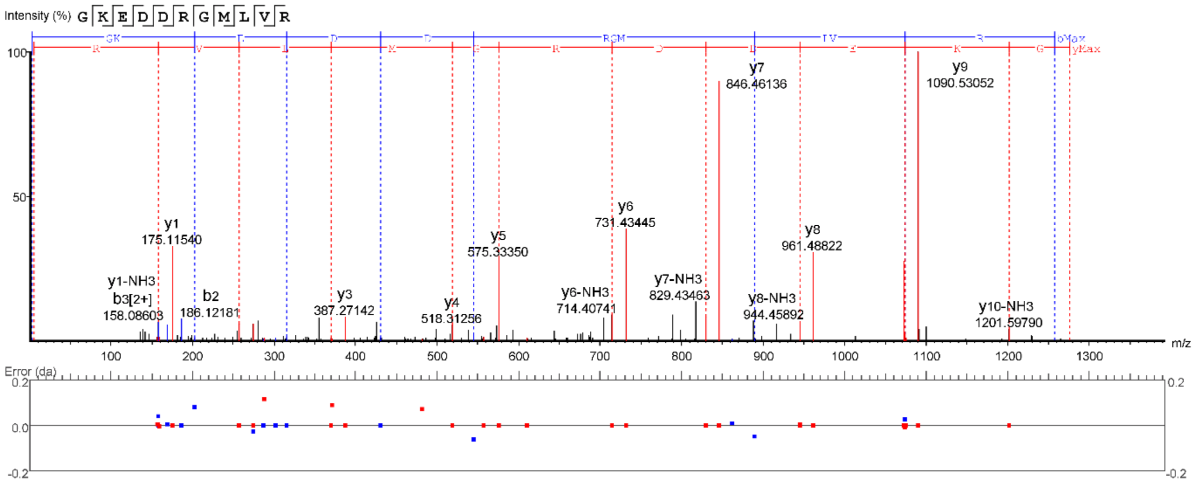

Intensity (%) G K E D D R G M L V R
y7
846.46136
y9
1090.53052
y6
731.43445
y1
175.11540
y5
575.33350
y8
961.48822
y1-NH3
b3[2+]
158.08603
b2
186.12181
y3
387.27142
y4
518.31256
y6-NH3
714.40741
y7-NH3
829.43463
y8-NH3
944.45892
y10-NH3
1201.59790
m/z
Error (da)

Se-peptides

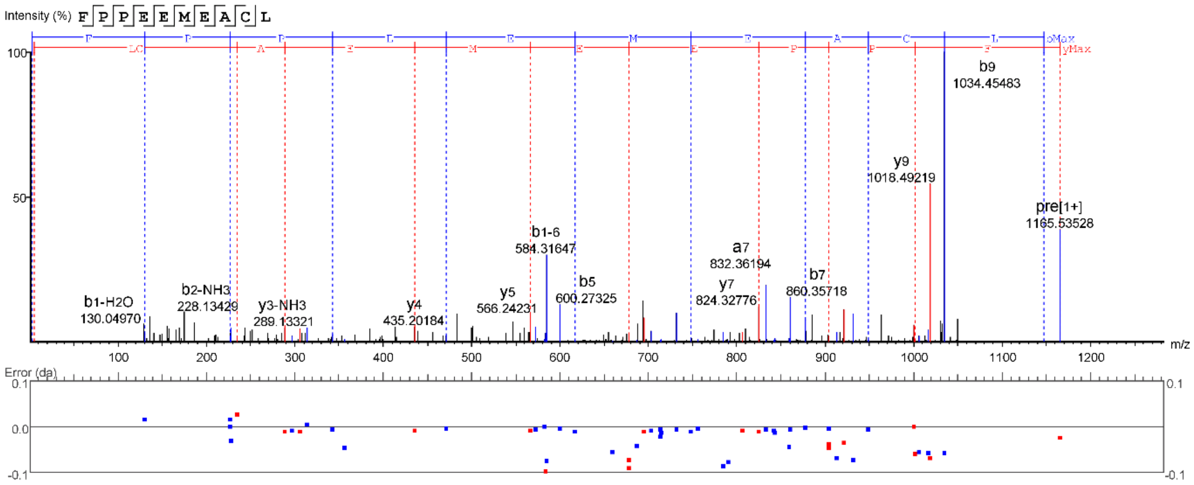

Intensity (%) F P P E E M E A C L
b9 1034.45483
y9 1018.49219
pre[1+] 1165.53528
b1-6 584.31647
a7 832.36194
b7 860.35718
y7 824.32776
b5 600.27325
y5 566.24231
y4 435.20184
y3-NH3 289.13321
b2-NH3 228.13429
b1-H2O 130.04970
m/z
Error (da)

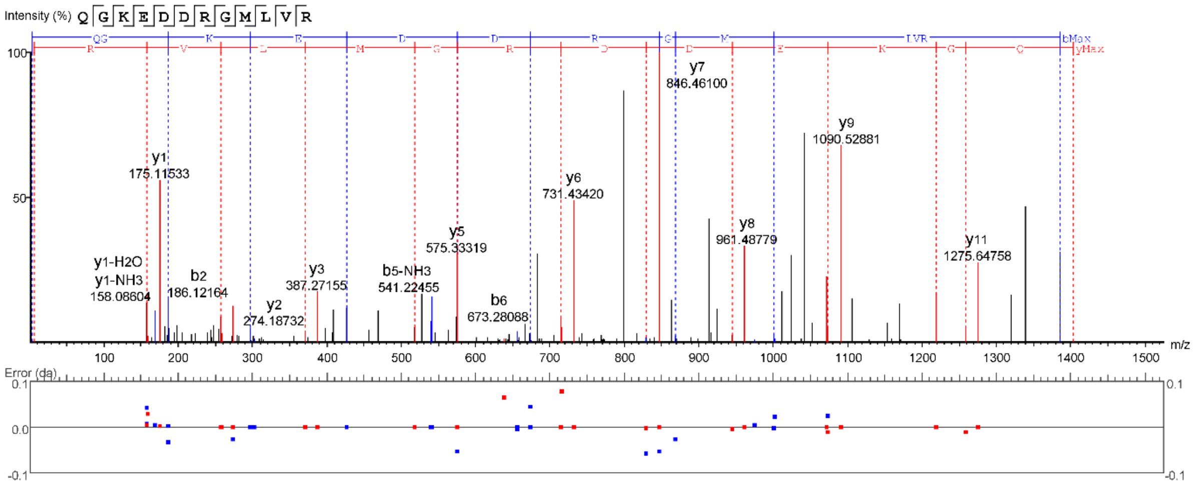

Intensity (%) Q G K E D D R G M L V R
y7 846.46100
y9 1090.52881
y1 175.11533
y6 731.43420
y8 961.48779
y11 1275.64758
y5 575.33319
y1-H2O
y1-NH3 158.08604
b2 186.12164
y3 387.27155
b5-NH3 541.22455
y2 274.18732
b6 673.28088
m/z
Error (da)

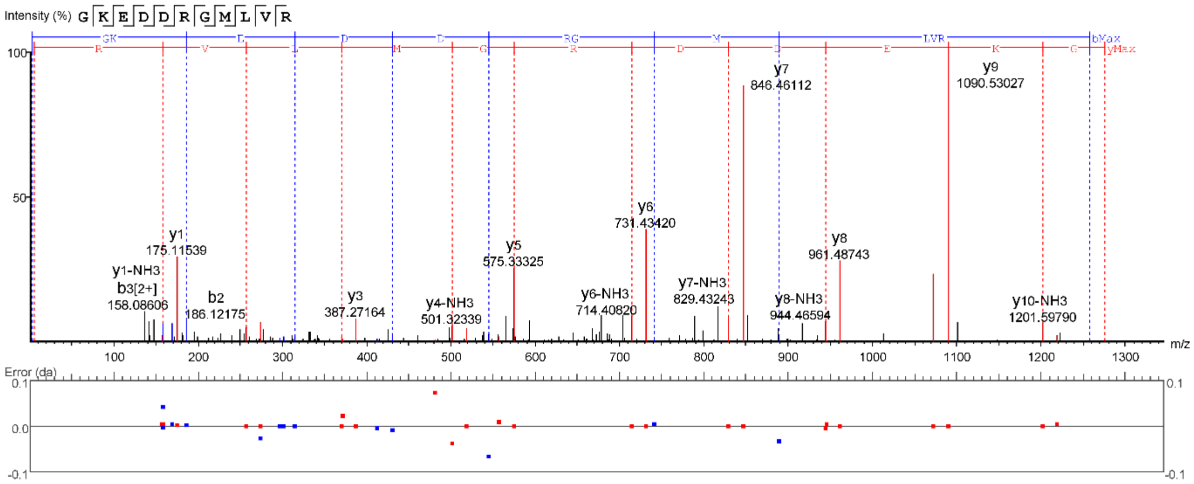

Intensity (%) G K E D D R G M L V R
y7 846.46112
y9 1090.53027
y6 731.43420
y1 175.11539
y5 575.33325
y8 961.48743
y1-NH3
b3[2+] 158.08606
b2 186.12175
y3 387.27164
y4-NH3 501.32339
y6-NH3 714.40820
y7-NH3 829.43243
y8-NH3 944.46594
y10-NH3 1201.59790
m/z
Error (da)

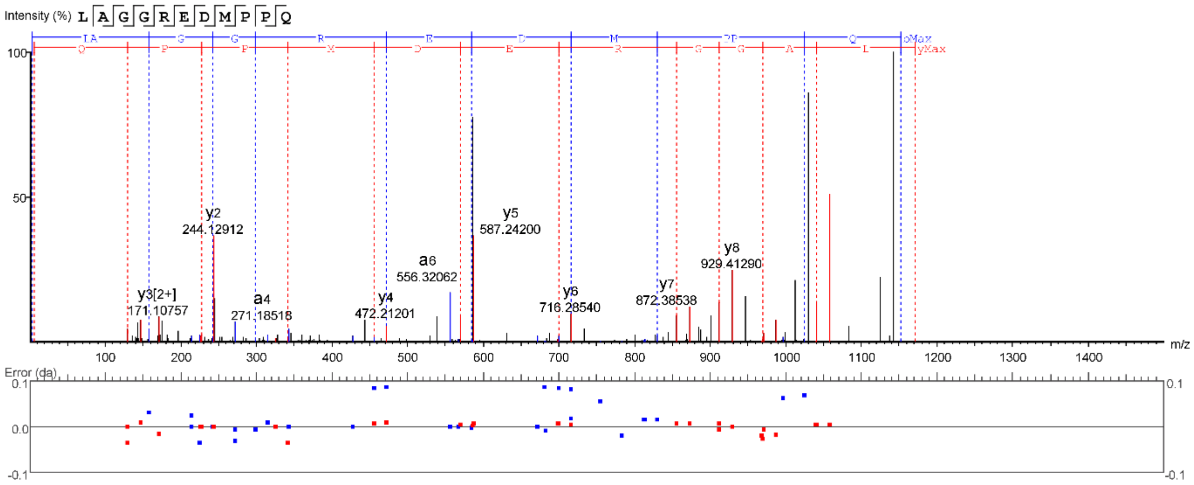

Intensity (%) L A G G R E D M P P Q
y2 244.12912
y3[2+] 171.10757
a4 271.18518
y4 472.21201
a6 556.32062
y5 587.24200
y6 716.28540
y7 872.38538
y8 929.41290
m/z
Error (da)

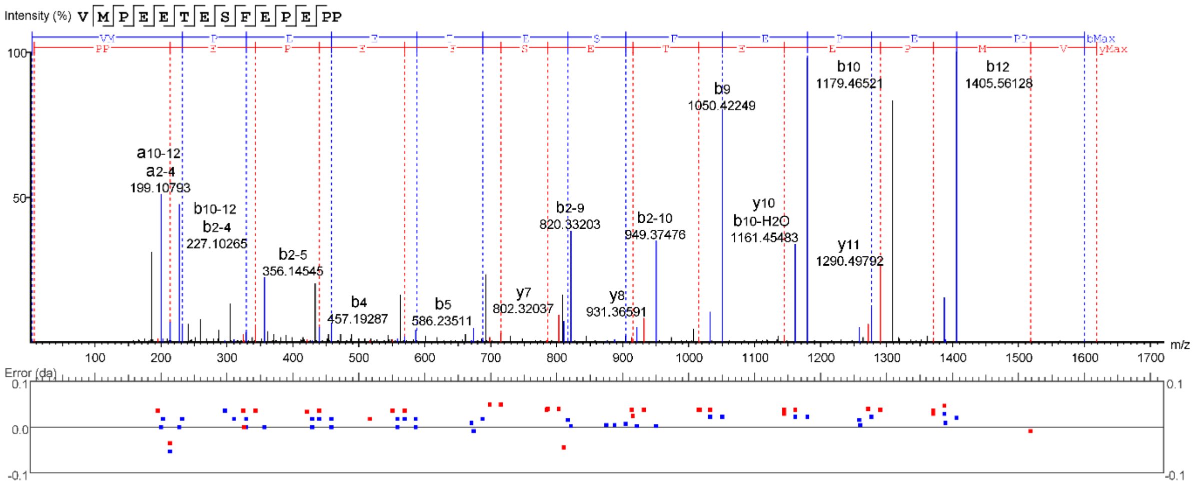

Intensity (%) V M P E E T E S F E P E PP
a10-12 a2-4 199.10793
b10-12 b2-4 227.10265
b2-5 356.14545
b4 457.19287
b5 586.23511
y7 802.32037
b2-9 820.33203
y8 931.36591
b2-10 949.37476
b9 1050.42249
y10 b10-H2O 1161.45483
b10 1179.46521
y11 1290.49792
b12 1405.56128
m/z
Error (da)

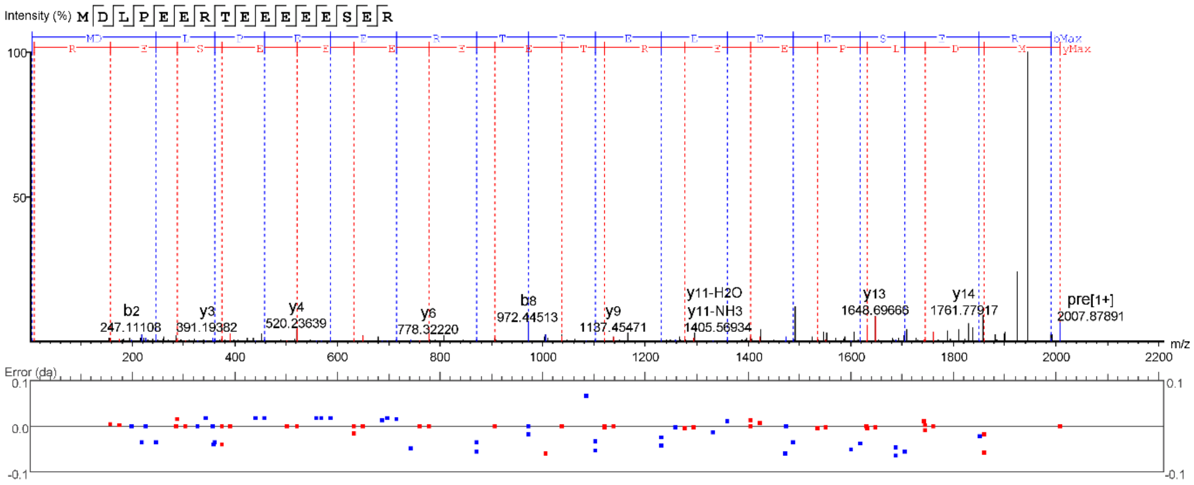

Intensity (%) M D L P E E R T E E E E S E R
b2 247.11108
y3 391.19382
y4 520.23639
y6 778.32220
b8 972.44513
y9 1137.45471
y11-H2O y11-NH3 1405.56934
y13 1648.69666
y14 1761.77917
pre[1+] 2007.87891
m/z
Error (da)

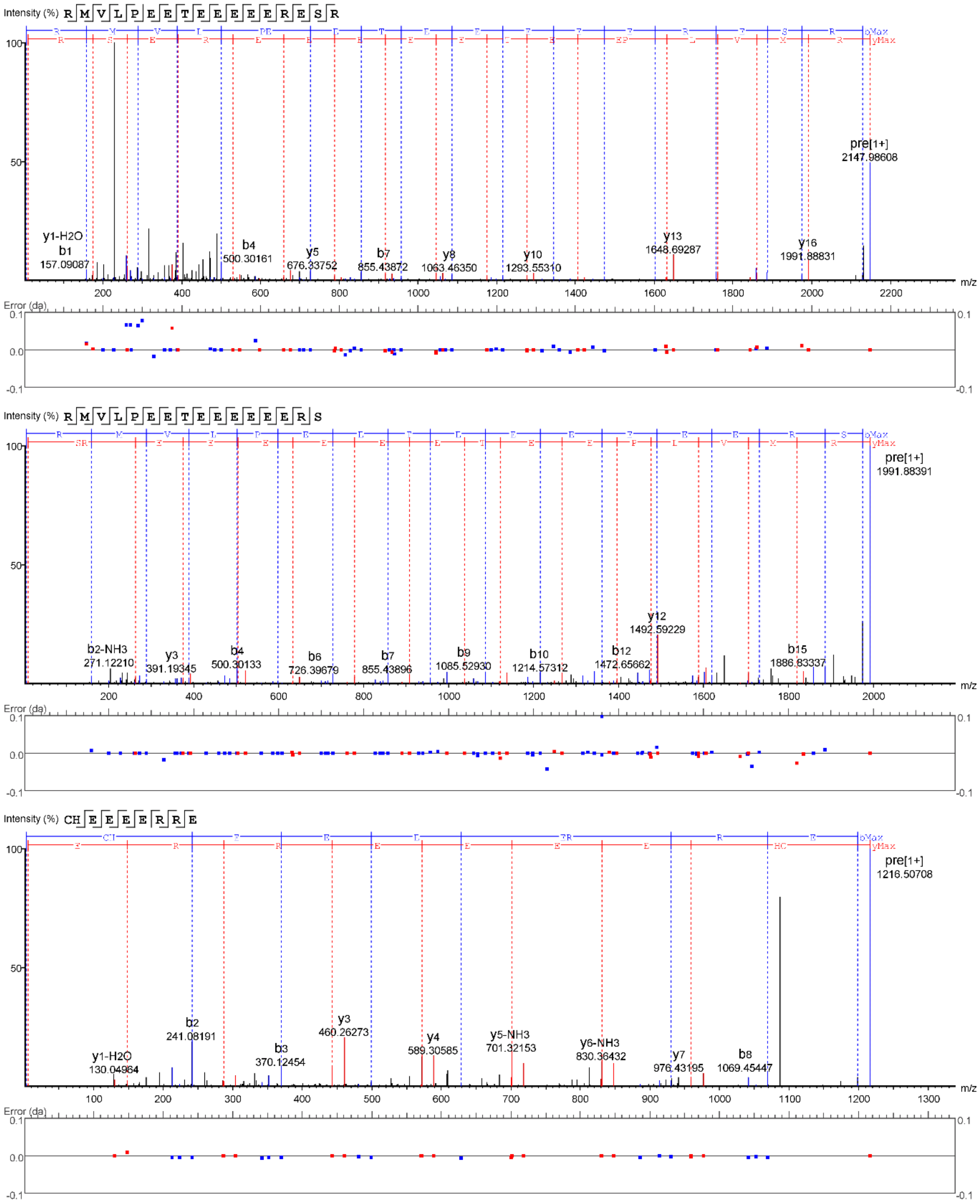

Fig. S2 MS/MS spectrum of bioactive peptides and selenopeptides from Se-enriched perilla seeds.
